# LIN-3/EGF Promotes the Programmed Cell Death of Specific Cells in *Caenorhabditis elegans* by Transcriptional Activation of the Pro-apoptotic Gene *egl-1*

**DOI:** 10.1371/journal.pgen.1004513

**Published:** 2014-08-21

**Authors:** Hang-Shiang Jiang, Yi-Chun Wu

**Affiliations:** 1 Institute of Molecular and Cellular Biology, National Taiwan University, Taipei, Taiwan; 2 Department of Life Science, National Taiwan University, Taipei, Taiwan; 3 Center for Systems Biology, National Taiwan University, Taipei, Taiwan; 4 Research Center for Developmental Biology and Regenerative Medicine, National Taiwan University, Taipei, Taiwan; 5 Institute of Atomic and Molecular Sciences, Academia Sinica, Taipei, Taiwan; University of Dundee, United Kingdom

## Abstract

Programmed cell death (PCD) is the physiological death of a cell mediated by an intracellular suicide program. Although key components of the PCD execution pathway have been identified, how PCD is regulated during development is poorly understood. Here, we report that the epidermal growth factor (EGF)-like ligand LIN-3 acts as an extrinsic signal to promote the death of specific cells in *Caenorhabditis elegans*. The loss of LIN-3 or its receptor, LET-23, reduced the death of these cells, while excess LIN-3 or LET-23 signaling resulted in an increase in cell deaths. Our molecular and genetic data support the model that the LIN-3 signal is transduced through LET-23 to activate the LET-60/RAS-MPK-1/ERK MAPK pathway and the downstream ETS domain-containing transcription factor LIN-1. LIN-1 binds to, and activates transcription of, the key pro-apoptotic gene *egl-1*, which leads to the death of specific cells. Our results provide the first evidence that EGF induces PCD at the whole organism level and reveal the molecular basis for the death-promoting function of LIN-3/EGF. In addition, the level of LIN-3/EGF signaling is important for the precise fine-tuning of the life-versus-death fate. Our data and the previous cell culture studies that say EGF triggers apoptosis in some cell lines suggest that the EGF-mediated modulation of PCD is likely conserved in *C. elegans* and humans.

## Author Summary

Programmed cell death (PCD) is an evolutionarily conserved cellular process that is important for metazoan development and homeostasis. The epidermal growth factor (EGF) promotes cell proliferation, differentiation and survival during animal development. Surprisingly, we found that the EGF-like ligand LIN-3 also promotes the death of specific cells in *Caenorhabditis elegans*. We found that the LIN-3/EGF signal can be secreted from a cell to facilitate the demise of cells at a distance by activating the transcription of the PCD-promoting gene *egl-1* in the doomed cells through the transcription factor LIN-1. LIN-1 binds to the *egl-1* promoter *in vitro* and is positively regulated by the LIN-3/EGF, LET-23/EGF receptor, and the downstream MAPK signaling pathway. To our knowledge, LIN-3/EGF is the first extrinsic signal that has been shown to regulate the intrinsic PCD machinery during *C. elegans* development. In addition, the transcription factor LIN-31, which binds to LIN-1 and acts downstream of LIN-3/EGF, LET-23/EGF receptor, and the MAPK signaling pathway during vulval development, is dispensable for PCD. Thus, LIN-3/EGF promotes cell proliferation, differentiation, and PCD through common downstream signaling molecules but acts via distinct sets of transcription factors for different target gene expression.

## Introduction

PCD is important for proper animal development and tissue homeostasis [1], [2] and its dysregulation can cause aberrant death or survival of cells, which may lead to developmental defects, degenerative diseases, or cancers [1], [2].

*Caenorhabditis elegans* is an excellent model for studying PCD because of its invariant cell lineage and the conserved cell death pathway [3], [4]. Throughout the development of the *C. elegans* adult hermaphrodite, 131 somatic cells undergo PCD in an essentially invariant temporal and spatial pattern [5], [6]. Genetic and molecular studies have identified four genes, *egl-1* (BH3-only gene), *ced-9* (*bcl-2*), *ced-4* (*apaf-1*), and *ced-3* (caspase), that function in the core PCD pathway [7]–[12]. In living cells, CED-9 interacts with, and sequesters, CED-4 at the surface of mitochondria to prevent the cells undergoing PCD [13]. In cells destined to die, EGL-1 binds to CED-9, resulting in a conformational change in CED-9 and the release of bound CED-4 [14]. The released CED-4 translocates from the mitochondrion to the perinuclear membrane and interacts with, and activates, the caspase CED-3, leading to the eventual demise of the cell [15]. A recent study in mid-embryos and the germline suggested the existence of an alternative cell death activation mechanism that does not involve a direct interaction between CED-4 and CED-9 [16]. The transcriptional regulation of *egl-1* is a critical step in the induction of most PCD events in the embryo [17]. Several transcription factors controlling *egl-1* transcription have been identified and shown to specify the PCD fate of specific cells [4], [18]. For example, two transcription factors HLH-2 and HLH-3 activate *egl-1* transcription by direct binding to the *egl-1* cis-regulatory region during the specification of the death fate of NSM sister cells [18], [19]. Like HLH-2 and HLH-3, cell death specification genes have been shown to transcriptionally regulate the components of the core PCD machinery in a cell-autonomous manner. It is unclear whether the PCD fate, like many other cell fates, may be regulated by an extrinsic signal.

Extrinsic signals are crucial for a variety of developmental processes and act through receptors to elicit specific biological functions in a cell-nonautonomous manner. One example of such a signal-receptor pair is epidermal growth factor (EGF) and the EGF receptor (EGFR), which are involved in cell proliferation, differentiation, migration, survival, and death [20]–[22]. EGF has long been considered as a growth factor, since it stimulates proliferation in cultured cells, animals, and humans [23]. It also protects cells from apoptosis, as shown in cultured cells and Drosophila [24]–[29]. EGF can signal through the RAS-ERK-mediated and/or PI3K-mediated pathway(s) to activate transcription of various anti-apoptotic proteins, such as Bcl-X_L_ and Mcl-1 [25]–[27], and also regulates post-transcriptional modifications, such as phosphorylation of BAD and caspase-9, to prevent apoptosis [28], [29]. However, in contrast to this cytoprotective function, it has also been shown to promote apoptosis, as exogenous EGF induces apoptosis in several cell lines, such as A431, MDA-MB-468, and MCF-7 [30]–[32]. It is not clear how it exerts different functions in different cells under different conditions or whether the apoptosis-promoting function plays a physiological role during animal development.

In *C. elegans*, *lin-3* and *let-23* encode, respectively, the sole EGF-like ligand and the EGFR and control many aspects of development, including ovulation, vulval differentiation, cell specification, and behavioral quiescence [33]–[38]. Activated LET-23 can recruit SEM-5 (orthologous to human Grb2) from the cytosol to the plasma membrane to activate LET-60, a member of the GTP-binding RAS family [39], [40]. LET-60 then triggers a kinase cascade involving the sequential phosphorylation of LIN-45 (a Raf ortholog), MEK-2 (a MAPK kinase kinase), and MPK-1 (an ERK ortholog) [41]–[45]. Once MPK-1 is phosphorylated, it translocates to the nucleus and regulates the transcription of numerous target genes by phosphorylation of specific transcription factors [46]. Besides activating the LET-60-MPK-1 pathway, LET-23 activates the PLC-γ-mediated signaling pathway to regulate ovulation and behavioral quiescence [36], [38]. In addition, the SEM-5-binding protein SOC-1 is involved in both the PLC-γ- and PI3K-mediated signaling pathways [47]. The LET-60-MPK-1- and PI3K-mediated signaling pathways have been shown to be involved in physiological and genotoxic stress-induced germline cell death [48]–[51]. However, how these pathways are regulated by upstream signals, how they are linked to the intrinsic PCD machinery to control germline cell death, and whether they play a role in somatic PCD remain unknown.

We now report that LIN-3 acts as an extrinsic signal to promote specific somatic PCDs and the identification of components that transmit the LIN-3 signal to the intrinsic PCD machinery. Our data indicate that secreted LIN-3 induces cell death through the receptor LET-23, which acts in a cell-autonomous manner during PCD. Our genetic and biochemical data suggest that the LET-60-MPK-1 pathway transmits the LIN-3 signal from LET-23 to the transcription factor LIN-1 for transcriptional up-regulation of *egl-1*, which leads to activation of PCD.

## Results

### *lin-3* and *let-23* promote specific PCDs in *C. elegans*

**Figure 1:**
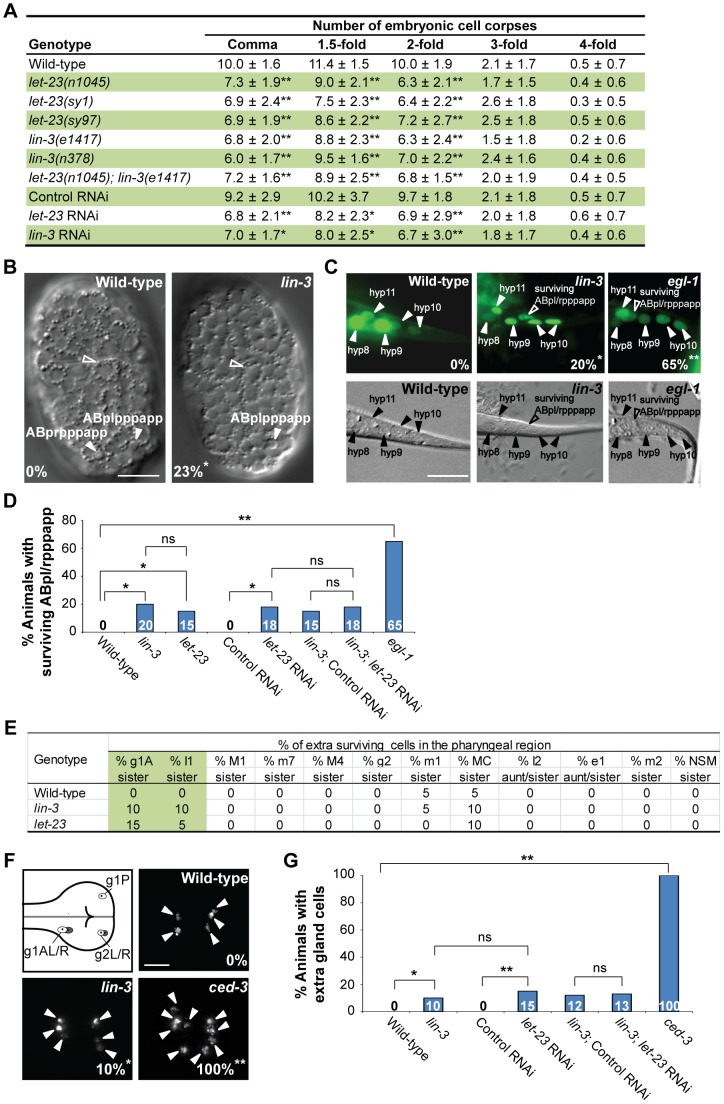
*lin-3* and *let-23* promote specific PCDs in *C. elegans*. (A) The number of cell corpses was scored at indicated developmental stages and presented as the mean ± SD (n≥30). The *rrf-3(pk1426)* mutant was used to enhance the RNAi effect in RNAi experiments. Mutants were compared to wild-type, and RNAi treatment of individual genes was compared to control RNAi (*P<0.05 and **P<0.001, two-tailed *t* test). (B) The DIC images of the wild-type and *lin-3(e1417)* mutant embryos at approximately 270 min after fertilization. The white arrowheads indicate ABpl/rpppapp cell corpses and the hollow arrowheads indicate excretory cells. The percentage of embryos that did not have ABpl/rpppappcell corpse is shown in the bottom left corner. *P = 0.048 when compared to wild-type (Fisher's exact test). Twenty one and thirteen embryos were scored for wild-type and *lin-3* mutants, respectively. Scale bar: 10 µm. (C) The GFP and DIC images of the wild-type, *lin-3(e1417)*, and *egl-1(n1084n3082)* worms expressing *sur-5::gfp* at the L1 stage. The arrowheads indicate the nuclei of hyp8, hyp9, hyp10, and hyp11. The hyp10 cell is binuclear. The hollow arrowheads indicate a surviving ABpl/rpppapp. The percentage of worms with surviving ABpl/rpppapp is shown in the bottom right corner. *indicates *P*<0.05 and **P<0.001 when compared to wild-type (Fisher's exact test). More than thirty worms were scored for each genotype. Scale bar: 10 µm. The GFP protein expressed from the *sur-5::gfp* transgene is localized to almost all somatic nuclei [86]. (D) The percentage of worms with surviving ABpl/rpppapp of the indicated genotypes was shown. *indicates *P*<0.05 and **P<0.001 (Fisher's exact test). ns indicates no statistical difference (p>0.05). Alleles used here are *lin-3(e1417)*, *let-23(sy1)*, and *egl-1(n1084n3082)*. More than thirty worms were scored for each genotype. (E) Analysis of surviving cells in the pharyngeal region (n = 20). Alleles used here are *lin-3(e1417)* and *let-23(n1045)*. (F) The g1 and g2 gland cells in the pharynx are shown in the illustration. The white circles indicate the five gland cells (g1P, g1AL, g1AR, g2L, and g2R) that normally survive and the gray circles the sister cells of g1AL, g1AR, g2L, and g2R that are destined to die. Monomeric red fluorescent protein (mRFP) images of the wild-type, *lin-3(e1417)*, and *ced-3(n717)* worms expressing *P_B0280.7_::4Xnls::mrfp* are shown. The arrowheads indicate cells expressing mRFP. The percentage of animals that have extra gland cell(s) is shown in the bottom right corner. *indicates *P*<0.05 and **P<0.001 when compared to wild-type (Fisher's exact test). One hundred L4 animals were scored for each genotype. The image stacks were merged by maximum intensity projection using Image J. Scale bar: 10 µm. (G) The percentage of worms with extra gland cells of the indicated genotypes was shown. *indicates *P*<0.05 and **P<0.001 (Fisher's exact test). ns indicates no statistical difference (p>0.05). Alleles used here are *lin-3(e1417)* and *ced-3(n717)*. More than forty worms were scored for each genotype.

While screening to test the potential involvement of receptor tyrosine kinase genes in PCD (see Materials and Methods), we found that several *let-23* mutants have reduced numbers of cell corpses (Figure 1A). The *let-23* gene encodes EGFR [34], raising the intriguing possibility that LET-23 might mediate an outside-in signal to affect PCD. *lin-3* encodes the only EGF in *C. elegans* [33] and we found that *lin-3* mutants had a similar reduction in numbers of cell corpses as *let-23* single mutants or the *let-23; lin-3* double mutant at the same embryonic stages (Figure 1A). This shows that *lin-3* and *let-23* function in the same pathway to affect cell corpse number, probably by acting as a ligand-receptor pair. Inactivation of *lin-3* or *let-23* by RNA interference (RNAi) reduced the numbers of cell corpses to a similar extent as the *lin-3* or *let-23* mutations (Figure 1A). Because the *lin-3* or *let-23* RNAi treatment caused more than 50% rod-like larval lethality, phenocopying the strong loss-of-function *lin-3* or *let-23* mutants [52], the reduced numbers of cell corpses observed in the *lin-3* or *let-23* mutants mentioned above likely represented the phenotype caused by nearly complete elimination of *lin-3* or *let-23*, respectively. Interestingly, in these mutants, only a partial reduction of cell corpse numbers from the comma stage to the 2-fold stage was observed, suggesting that *lin-3* and *let-23* may be essential for some but not all cell deaths.

To investigate whether *lin-3* may affect specific cell deaths, we examined the first 13 cell deaths that occur in the AB lineage during embryogenesis [6], [53] in the *lin-3(e1417)* mutant by using four-dimensional DIC microscopy. In three out of thirteen *lin-3* embryos we analyzed, only ABplpppapp or its lineal homolog ABprpppapp failed to die, and the other cell deaths occurred normally (Figure 1B and S1). ABplpppapp and ABprpppapp (these cells are collectively termed ABpl/rpppapp hereafter), which are aunt cells of hypodermal cells hyp8/9, are generated at approximately 270 min after fertilization and undergo PCD soon after birth [6]. Consistent with the DIC four-dimensional microscopy data, approximately 20% of surviving ABpl/rpppapp were observed in the tail of the *lin-3(e1417)* mutant (Figure 1C and D), indicating that the disappearance of ABpl/rpppapp corpses is due to the survival of these cells. A similar result was observed in the *let-23(sy1)* mutant and in the *lin-3(e1417)* mutant feeding *let-23* RNAi (Figure 1D), showing that *lin-3* and *let-23* act in the same pathway to promote the death of ABpl/rpppapp.

We also measured the duration time of the first 13 cell corpses derived from the AB lineage in the wild-type and *lin-3(e1417)* mutant embryos and found that the corpse duration time was statistically undistinguishable in the wild-type and *lin-3* embryos (Figure S2, *P* = 0.24, two-tailed *t* test). This result indicates that the kinetics of corpse removal is normal in the *lin-3(e1417)* mutant, and rules out the possibility that the decrease of cell corpse numbers in the *lin-3* mutants may be attributed to enhanced cell corpse removal.

We next examined whether *lin-3* or *let-23* may also be important for the death of other PCDs. We scored the extra surviving cells in the pharynx of the *lin-3* or *let-23* mutant at the L3 and L4 stages. In wild-type animals, 16 cells in the anterior pharynx and 6 in the posterior pharynx undergo PCD during mid-embryogenesis. Eight out of these 22 cells (NSML/R, I2L/R, g1AL/R and g2L/R) undergo PCD during the comma to 2-fold stage. In virtually all wild-type embryos, no extra surviving cells are observed in these regions at the late larval stages [6], [11], [54]. In *lin-3* and *let-23* mutants, about 10% of the sister cells of g1A gland cells and I1 interneurons survived, and the death of other 18 cells in the pharynx region was essentially normal (Figure 1E). Furthermore, the percentage of animals with surviving g1A and I1 sisters was enhanced by the weak *ced-3(n2427)* mutation; however, essentially no enhancement was observed in other 18 cells examined (Table S1). This result shows that *lin-3* and *let-23* are important for the death of specific cells. Consistently, using an integrated transgene *tpIs8*, which expresses monomeric red fluorescent protein (mRFP) in the g1A, g1P, and g2 gland cells [55], we found that 10% of the *lin-3* animals had extra gland cells, whereas wild-type worms had no extra gland cell and the strong *ced-3(n717)* mutants all had extra gland cell(s) (Figure 1F and G). This result indicates that, like *ced-3*, surviving cells in the *lin-3* mutant adopt the terminal differentiation fate of their sister cells. A similar result was observed in the wild-type or *lin-3(e1417)* mutant feeding *let-23* RNAi (Figure 1G), showing that *lin-3* and *let-23* also act in the same pathway to promote the death of gland cells. Our data show that *lin-3* and *let-23* are important for the death of specific cells, including g1A and I1 sister cells and ABpl/rpppapp and that these genes are dispensable for most cell deaths analyzed in this work.

### Overexpression of *lin-3* cause ectopic cell deaths

**Figure 2:**
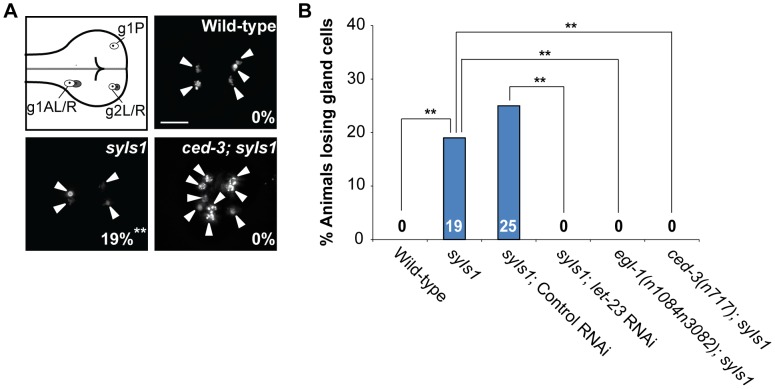
Overexpression of *lin-3* induces ectopic gland cell death(s) through *let-23* and the core PCD pathway. (A) Monomeric red fluorescent protein (mRFP) images of the wild-type, *syIs1*, and *ced-3(n717); syIs1* worms expressing *P_B0280.7_::4Xnls::mrfp* are shown. The arrowheads indicate cells expressing mRFP. The percentage of animals losing gland cell(s) is shown in the bottom right corner. **indicates *P*<0.001 when compared to wild-type (Fisher's exact test). One hundred L4 animals were scored for each genotype. The image stacks were merged by maximum intensity projection using Image J. Scale bar: 10 µm. (B) The percentage of worms losing gland cell(s) of the indicated genotypes was shown. **indicates *P*<0.001 (Fisher's exact test). More than forty worms were scored for each genotype.

We next examined whether over-activation of *lin-3* signaling could cause ectopic cell deaths during embryogenesis. We found that overexpression of *lin-3* genomic DNA from the integrated transgene *syIs1* [33] increased embryonic cell corpse numbers (Table S2). Using the integrated transgene *tpIs8* to label g1 and g2 gland cells, we found that *syIs1* caused the loss of one or two gland cells in 19% of transgenic worms (Figure 2A and B). This phenotype of gland cell loss was suppressed by the loss-of-function mutation in *egl-1* or *ced-3* (Figure 2A and B). This result shows that *lin-3* overexpression induces ectopic gland cell death rather than alters gland cell fate, and that the cell death-promoting activity of *lin-3* requires the core PCD pathway. In addition, the phenotype of ectopic gland cell death induced by *syIs1* was also suppressed by *let-23* RNAi (Figure 2B), showing that *lin-3* acts through *let-23* to promote the ectopic cell death of the gland cells. Consistently, the increased numbers of embryonic cell corpses induced by *syIs1* was also suppressed by loss-of-function mutations in *let-23* or in components of the core PCD pathway (Table S2).

### *let-23* is expressed in dying cells, including ABpl/rpppapp

**Figure 3:**
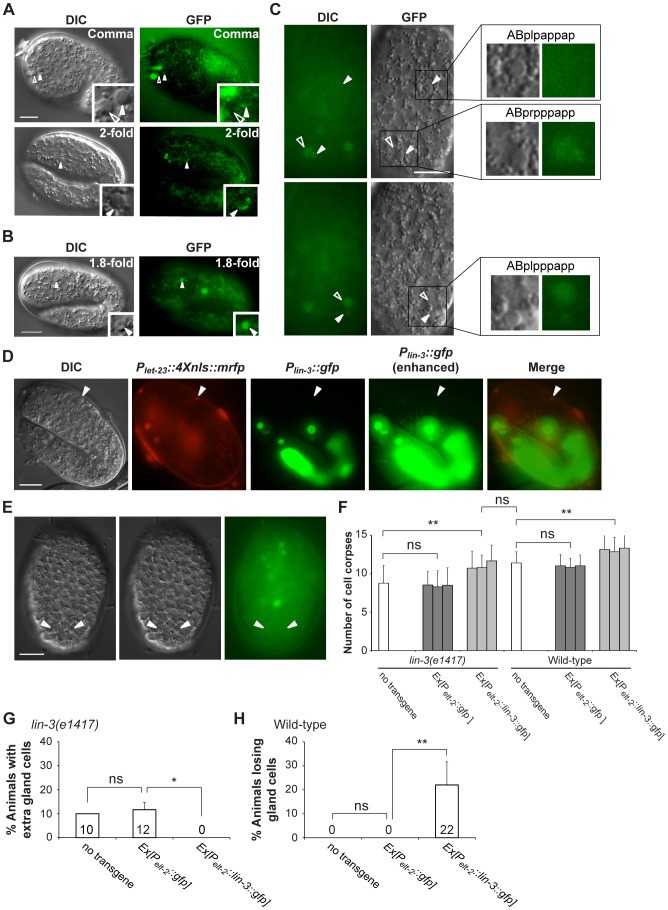
*let-23* is expressed in dying cells, whereas *lin-3* acts in a cell-nonautonomous manner to promote PCD. (A and B) Representative DIC and GFP images of embryos carrying (A) the *syEx234[P_let-23_::let-23::gfp]* transgene or (B) the *P_let-23_::4Xnls::gfp* transgene. The white arrowheads indicate cell corpses expressing LET-23::GFP and the hollow arrowheads cell corpses not expressing LET-23::GFP. Scale bar: 10 µm. The insets show a dying cell at a three-fold higher magnification. (C) The DIC and GFP images of an embryo carrying the *P_let-23_::4Xnls::gfp* transgene. Two different focal planes of the same embryo are shown in the upper and lower panels. The white arrowheads indicate ABpl/rpppapp corpses and ABplpappap and the hollow arrowheads sister cells of ABpl/rpppapp. Scale bar: 10 µm. The insets show the indicated cells at a higher magnification. (D) The expression pattern of *P_lin-3_::gfp* and *P_let-23_::4Xnls::mrfp* transgenes in a wild-type embryo. The white arrowheads indicate the same cell corpse expressing *P_let-23_::4Xnls::mrfp*. The image stacks of *P_lin-3_::gfp* were merged by maximum intensity projection using Image J. The strong GFP expression is from the co-injection marker *P_elt-2_::gfp*. Scale bar: 10 µm. (E) *P_lin-3_::gfp* is not expressed in ABpl/rpppapp corpses. The image stacks of *P_lin-3_::gfp* were merged by maximum intensity projection using Image J. Scale bar: 10 µm. (F–H) Intestine-specific expression of LIN-3 rescues the cell death defect of the *lin-3* mutant and causes ectopic cell deaths in the wild-type. Embryonic cell corpses in the indicated genotypes were scored at the 1.5-fold stage (n≥30), and the numbers of gland cells were scored at the L4 stage (n≥40). Three independent stable transgenic lines were analyzed. *indicates *P*<0.05 and **P<0.001 in a two-tailed *t*-test (F) or fisher's exact test (G and H). ns indicates no statistical difference (p>0.05).

Next, we examined whether there was a correlation between *let-23* expression and PCD using the transgene *syEx234[P_let-23_::let-23::gfp]* [56]. As shown in Figure 3A, the LET-23::GFP fusion protein was detected as a crescent on the surface of some dying cells (indicated by arrowheads). The cause and physiological significance of this crescent-shaped localization pattern is not clear. To be sure that *let-23* was expressed in dying cells and not on the membranes of adjacent living cells, we generated the construct *P_let-23_::4Xnls::gfp* and microinjected it into the wild-type to generate transgenic worms. *P_let-23_::4Xnls::gfp* contains the *let-23* promoter *P_let-23_* fused to the GFP cDNA containing four copies of the nuclear localization signal (4XNLS). The GFP signal was detected in the nuclei of the dying cells (Figure 3B), clearly showing that *let-23* is expressed in dying cells. Notably, the GFP signal was observed in the ABpl/rpppapp corpses (Figure 3C).

### LIN-3 can act as an extrinsic signal to promote PCD

Like its *Drosophila* and vertebrate homologs, *lin-3* encodes a transmembrane precursor protein, consisting of an extracellular domain containing one EGF motif, a transmembrane domain, and a cytoplasmic domain [33], [57]. Genetic and laser ablation studies have suggested that the LIN-3 precursor protein can be cleaved to form a secreted product [33], [58], [59]. Using the transcriptional reporter *P_lin-3_::gfp* [60] and *P_let-23_::4Xnls::mrfp*, no *P_lin-3_::gfp*-expressing cell was located adjacent to the *P_let-23_::4xnls::rfp*-expressing cell corpses, as shown in Figure 3D. In addition, no *P_lin-3_::gfp* signal was detected near ABpl/rpppapp corpses (Figure 3E). These observations raise a possibility that *lin-3* may be secreted and act at a distance to promote PCD.

To test this hypothesis, we generated a DNA construct *P_elt-2_::lin-3::gfp*, in which a *lin-3::gfp* fusion cDNA was expressed under the control of the *elt-2* promoter *P_elt-2_* and introduced it to the wild-type and *lin-3* mutant. *P_elt-2_* is used here because it is exclusively expressed in intestinal cells and their precursor cells [61] and no PCD occurs in these cells [6]. We found that, in the *lin-3* mutant, the *P_elt-2_::lin-3::gfp* transgene not only increased the number of embryonic cell corpses to the wild-type level (Figure 3F) but also rescued the inappropriate survival of gland cells (Figure 3G). Moreover, when introduced to the wild-type, the *P_elt-2_::lin-3::gfp* transgene increased the embryonic cell corpse number (Figure 3F) and caused ectopic gland cell deaths (Figure 3H), as the transgene *syIs1* did (Figure 2A, B and Table S2). These results show that LIN-3::GFP secreted from the intestine can act at a distance to induce cell death in other parts of the embryo, e.g. gland cells in the head region. In our study, the adult worms transgenic for *P_elt-2_::lin-3::gfp* had a multi-vulva phenotype, resembling worms carrying *syIs1* [33], reinforcing the notion that LIN-3::GFP expressed in the intestine is secreted and active.

### *lin-3* signaling up-regulates *egl-1* transcription

**Figure 4:**
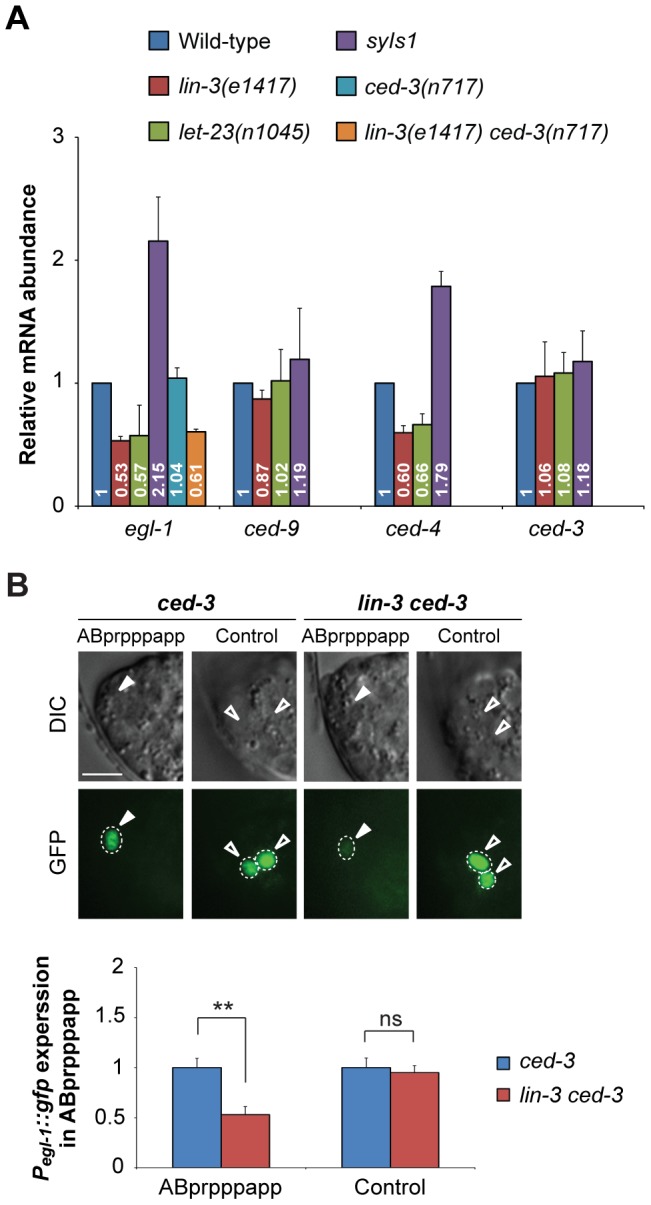
*lin-3* promotes *egl-1* transcription. (A) *egl-1*, *ced-9*, *ced-4*, and *ced-3* transcripts in embryos of indicated genotypes were measured by quantitative RT-PCR. The relative mRNA abundance is shown as the mean and SD of the results from at least two independent experiments, each performed in triplicates. (B) The DIC and GFP images of *ced-3* (left) and *lin-3 ced-3* (right) embryos expressing the *P_egl-1_::gfp* transgene. Arrowheads indicate ABprpppapp, and hollow arrowheads indicate two cells that also express *egl-1* near ABprpppapp (Control experiment). The bottom figures show the *P_egl-1_::gfp* expression level of the indicated cells in the *ced-3* single mutant (blue) and the *lin-3 ced-3* double mutant (red). The expression level of *P_egl-1_::gfp* in ABprpppapp of *ced-3(n717)* (n = 44) and *lin-3(e1417) ced-3(n717)* (n = 40) mutants was analyzed as described in Materials and Methods. Error bars represent the SEM. **indicates *P*<0.001 (two-tailed *t* test). Scale bar: 5 µm.

Because the cell death-promoting activity of *lin-3* requires the core PCD pathway (Figure 2A, B, and Table S2), we next examined whether *lin-3* signaling promotes PCD by regulating the expression of *egl-1*, *ced-9*, *ced-4*, or *ced-3*. To this end, we measured the transcript levels of *egl-1*, *ced-9*, *ced-4*, or *ced-3* in worms with different levels of *lin-3* activity by quantitative real-time reverse transcription (RT)-PCR. As shown in Figure 4A, the abundance of *egl-1* transcripts correlated well with the level of *lin-3* activity, which, in turn, correlated well with the number of cell deaths. *egl-1* transcripts were less abundant in *lin-3* and *let-23* mutants than in wild-type animals (average of 0.53-fold and 0.57-fold, respectively), while *lin-3* overexpression using the integrated transgene *syIs1* caused an increase in *egl-1* transcript levels (average of 2.15-fold). Similarly, levels of the *ced-4* transcript, but not those of the *ced-9* and *ced-3* transcripts, also showed a correlation with the level of *lin-3* activity, although to a lesser extent than the *egl-1* transcript. These data show that LIN-3 and LET-23 signaling activates transcription of *egl-1* and, to a lesser extent, *ced-4, in vivo*. Interestingly, we found that the *lin-3(e1417)* mutation reduced the *egl-1* transcript level in the *ced-3(n717)* mutant (average of 0.61-fold) to a similar extent as the *lin-3(e1417)* mutation did in the wild-type (Figure 4A). This result supports the model that the LIN-3-mediated up-regulation of *egl-1* transcription occurs before *ced-3* activation during PCD.

Next, we examined the effect of *lin-3* on the *egl-1* transcription activity in ABprpppapp using the transcriptional fusion reporter *P_egl-1::_gfp* [62]. The *egl-1(n1084n3082)* mutation caused frequent survival of ABpl/rpppapp, showing that *egl-1* is required for the death of ABprpppapp (Figure 1C and D). We measured and compared the fluorescence intensity of GFP in ABprpppapp of the *ced-3* and *lin-3 ced-3* mutants. The *ced-3(n717)* mutation blocks the death of ABprpppapp, hence allowing the observation of the *P_egl-1_::gfp* transgene in the surviving ABprpppapp, and has no effect on the *lin-3*-mediated *egl-1* transcription (Figure 4A). In the *ced-3(n717)* mutant, ABprpppapp survived and expressed *P_egl-1::_gfp* at the comma stage (Figure 4B). The *lin-3(e1417)* mutation significantly decreased the intensity of *P_egl-1_::gfp* to about 0.53-fold in ABprpppapp of the *ced-3* mutant (Figure 4B, *P* = 0.0004), consistent with the result from quantitative real-time RT-PCR (Figure 4A). Notably, the *lin-3(e1417)* mutation did not affect the *egl-1* transcription in two adjacent cells that also express *egl-1* (Figure 4B, control experiment, *P* = 0.6839). Therefore, loss of *lin-3* reduces the *egl-1* transcription level in a cell-specific manner. These data and the aforementioned observation that the *egl-1(lf)* mutation blocked the *syIs1*-induced cell death (Figure 2A, B, and Table S2) together support the notion that *lin-3* signaling promotes the death of ABprpppapp by transcriptional activation of *egl-1*.

### LET-23 transduces the cell death-promoting signal via the LET-60-MPK-1 pathway

**Figure 5:**
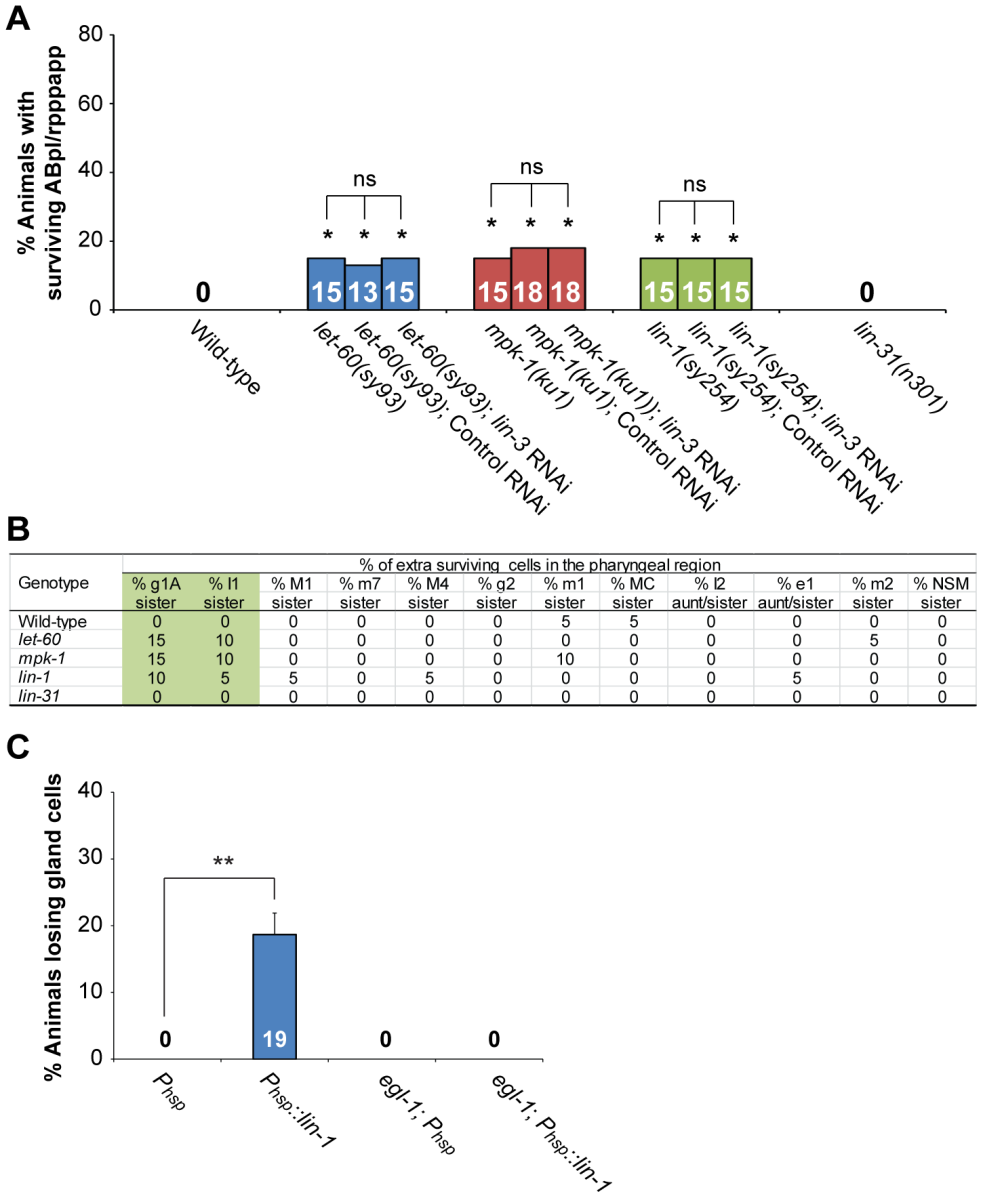
*lin-3*, *lin-1* and the LET-60-MPK-1 pathway act in the same pathway to promote PCD. (A) The percentage of worms with surviving ABpl/rpppapp of the indicated genotypes was shown. *indicates *P*<0.05 when compared to the wild-type (Fisher's exact test). ns indicates no statistical difference (p>0.05). More than thirty worms were scored for each genotype. (B) Analysis of extra surviving cells in the pharyngeal region (n = 20). Alleles used here are *let-60(sy93), mpk-1(ku1), lin-1(sy254), and lin-31(n301)*. (C) The percentage of wild-type or *egl-1(n1084n3082)* worms carrying the indicated transgene that lost gland cell(s) in the pharynx was shown. **P<0.001 (Fisher's exact test). The gland cells were scored using the *tpIs8* transgene, and the worms were treated with heat shock as described in Materials and Methods. *P_hsp_::lin-1* or *P_hsp_* indicate the transgenes, in which *lin-1* or no cDNA was expressed under the control of the heat-shock promoter, respectively. More than forty worms were scored for each genotype.

We next investigated how *lin-3* and *let-23* signaling may up-regulate *egl-1* to promote PCD. Activated LET-23 has been shown to act via multiple signaling pathways, including the LET-60-MPK-1- and PLC-γ-mediated pathways, to control *C. elegans* development [36], [38]–[45]. SOC-1, a SEM-5-binding protein, has been reported to act not only in the LET-60-MPK-1 pathway, but also in the PLC-γ- and PI3K-mediated signaling pathways [47]. We found that mutants defective in the LET-60-MPK-1 pathway had reduced cell corpse numbers during mid-embryogenesis (Table S3), while mutants defective in either the PI3K-mediated pathway (Table S4A) or PLC genes (Table S4B) had normal cell corpse numbers throughout embryogenesis. We further analyzed whether specific PCDs were affected in the *let-60* and *mpk-1* mutants using the assays described above. We found that *let-60* and *mpk-1* are required for the death of ABpl/rpppapp (Figure 5A), glA sisters, and I1 sisters (Figure 5B) but essentially dispensable for most cell deaths examined in this work. These data show that mutants defective in the LET-60-MPK-1 pathway have a PCD-defective phenotype similar to *lin-3* and *let-23* mutants. Furthermore, the *lin-3* RNAi treatment did not enhance the survival of ABpl/rpppapp in the *let-60* or *mpk-1* mutant (Figure 5A), showing that, at least in ABpl/rpppapp, *lin-3* and *let-23* transduce the cell death-promoting signal through the LET-60-MPK-1 pathway.

### LET-23 transduces the cell death-promoting signal via the transcription factor LIN-1, but not LIN-31

Two transcription factors, the ETS domain-containing protein LIN-1 and the WH domain-containing protein LIN-31, transduce LET-60-MPK-1 signaling to control vulval differentiation [63], [64]. In the absence of the LET-60-MPK-1 signaling pathway, LIN-1 forms a complex with LIN-31 and inhibits vulval induction [46]. Activation of the LET-60-MPK-1 pathway leads to phosphorylation of LIN-1 and LIN-31 by MPK-1, and phosphorylation of LIN-31 results in dissociation of LIN-1 and LIN-31 from each other and subsequent vulval induction [46]. Interestingly, the *lin-1(lf)* mutation also caused the survival of ABpl/rpppapp (Figure 5A), g1A sisters, and I1 sisters (Figure 5B), whereas the *lin-31(lf)* mutation did not (Figure 5A and B). These results show that *lin-1*, but not *lin-31*, affects specific PCD, despite both genes being necessary for vulval differentiation. *lin-1* mutants also displayed a reduction of embryonic cell corpse numbers to a similar extent as mutants defective in the LET-60-MPK-1 pathway, whereas *lin-31* mutants had a normal cell corpse profile during embryogenesis (Table S5). The *lin-3* RNAi treatment did not enhance the penetrance of ABpl/rpppapp survival in the *lin-1* mutant, showing that *lin-3* and *lin-1* act in the same pathway to promote the death of ABpl/rpppapp (Figure 5A). Consistently, the cell corpse numbers of the *lin-1(sy254) lin-3(e1417)* double mutant showed no significant difference when compared to the *lin-1(sy254)* or *lin-3(e1417)* single mutant (Table S5). These data and the aforementioned genetic results support the notion that LIN-3 and LET-23 act through the LET-60-MPK-1 pathway and transcription factor LIN-1 to promote PCD.

### LIN-1 activates *egl-1* transcription by direct binding to the *egl-1* promoter

We next studied the mechanism by which LIN-1 promotes PCD. As mentioned above, LIN-3 and LET-23 signaling activates transcription of *egl-1* and, to a lesser extent, *ced-4, in vivo* (Figure 4A). This was confirmed by examining the expression level of the transcriptional fusion reporter *P_egl-1_::gfp* in ABprpppapp (Figure 4B). Moreover, like *lin-3* overexpression, heat shock-induced *lin-1* overexpression in the wild-type caused the phenotype of gland cell loss, and this phenotype can be suppressed by an *egl-1(lf)* mutation (Figure 5C). These results support the model that LIN-1 acts upstream of *egl-1* to promote the death of gland cells.

**Figure 6:**
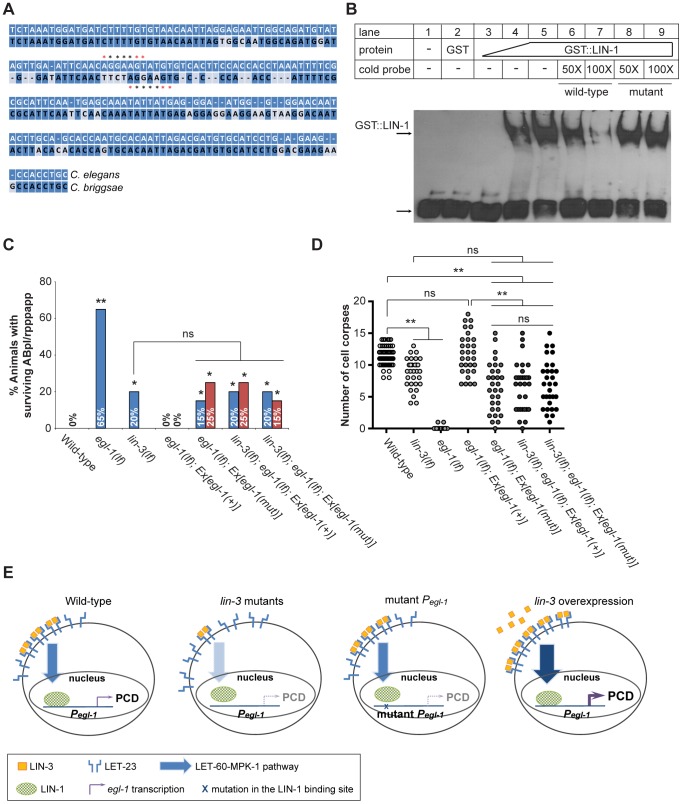
*lin-3* promotes PCD through transcriptional activation of *egl-1* by LIN-1. (A) The alignment between the *C. elegans* and *C. briggsae* genomic sequences near the P3 fragment. *indicates the LIN-1 minimal recognition sequence GGAA/T. (B) GST::LIN-1(DBD) directly binds to the *egl-1* promoter in an EMSA assay. Lane 1, no protein; lane 2, 200 ng of GST; lanes 3–5, 10, 50, or 200 ng of GST::LIN-1(DBD); lane 6: 200 ng of GST::LIN-1(DBD)+50× wild-type cold probe; lane 7, 200 ng of GST::LIN-1(DBD)+100× wild-type cold probe; lane 8, 200 ng of GST::LIN-1(DBD)+50× mutant cold probe; lane 9, 200 ng of GST::LIN-1(DBD)+100× mutant cold probe. The bottom arrow indicates non bound probe and the top arrow DNA bound to GST::LIN-1(DBD) and displaying retarded mobility. (C) The percentage of animals with surviving ABpl/rpppapp of the indicated genotypes was scored (n≥30). *Ex[egl-1(+)]* and *Ex[egl-1(mut)]* indicate wild-type and mutant *egl-1* transgenes, respectively, as described in the text. The surviving ABpl/rpppapp was scored using the *sur-5::gfp* reporter as described in Figure 1B. Two independent stably transmitted lines were analyzed for each transgene. Alleles used: *lin-3(e1417)* and *egl-1(n1084n3082)*. *indicates *P*<0.05 and **P<0.001 when *egl-1(lf)* and *lin-3(lf)* were compared to wild-type or the transgenic strains were compared to *egl-1(lf); Ex[egl-1(+)]* (Fisher's exact test). ns, no significance. (D) Cell corpses in the embryos of the indicated genotypes carrying the wild-type or mutant *egl-1* gene were scored at the 1.5-fold stage (n≥30). Each circle represents a single embryo. At least three independent stably transmitted lines were analyzed for each transgene. Alleles used: *lin-3(e1417)* and *egl-1(n1084n3082)*. **indicates *P*<0.001 in a two-tailed *t*-test. ns indicates no statistical difference (p>0.05). (E) Proposed model of how *lin-3* promotes PCD (see text for details).

LIN-1 contains a highly conserved ETS domain [64], which binds to DNA with the minimal recognition sequence GGAA/T [65]. A potential LIN-1 binding sequence was found in the promoter region of *egl-1*, but not that of *ced-4* (Figure 6A, see Materials and Methods for details), supporting the possibility that LIN-1 binds directly to *egl-1*. We therefore generated the GST::LIN-1(DBD) fusion protein, in which the DNA binding domain of LIN-1 was fused to glutathione S-transferase (GST), and tested its ability to bind to a 34 bp DNA fragment (named P3) containing the LIN-1 binding sequence in the electrophoretic mobility shift assay (EMSA). The results are shown in Figure 6B. The P3 fragment displayed similar mobility in the presence or absence of GST (lanes 1 and 2), confirming that GST does not bind to the DNA fragment. In contrast, the presence of GST::LIN-1(DBD) resulted in the dose-dependent appearance of a slowly migrating band (lanes 3–5), and this effect was eliminated by addition of an excess of non-labeled wild-type DNA competitor (lanes 6 and 7), but not with an excess of a DNA competitor with a mutation in the core GGAA/T motif (lanes 8 and 9). These results show that the slowly migrating band is a complex of GST::LIN-1(DBD) and P3 and that LIN-1 binds to the P3 region of *egl-1 in vitro*.

To test the physiological importance of the interaction between LIN-1 and the *egl-1* promoter, we mutated the P3 sequence in *egl-1* genomic DNA and assessed the ability of the mutant *egl-1* gene to rescue the cell death defect of the *egl-1* mutant. Specifically, we examined the cell death phenotype by scoring the surviving ABpl/rpppapp in the L1 tail (Figure 6C) and by counting the number of cell corpses at the 1.5-fold stage (Figure 6D). In the control experiment, *egl-1* mutants carrying the wild-type *egl-1* transgene showed no surviving ABpl/rpppapp (Figure 6C), indicating that the wild-type *egl-1* transgene *Ex[egl-1(+)]* fully rescued the *egl-1* cell death defect. When the LIN-1 recognition sequence CAGGAA in the P3 region of the *egl-1* promoter was mutated into GCTACT [65], the mutant *egl-1* transgene *Ex[egl-1(mut)]* only partially rescued the *egl-1* cell death defect. In the two transgenic lines, the percentages of ABpl/rpppapp survival were 15% and 25%, similar to that of the *lin-3* mutant (Figure 6C). The *egl-1; Ex[egl-1(mut)]* transgenic worms also had a similar number of embryonic cell corpses as the *lin-3* mutant at the 1.5-fold stage (Figure 6D). These results show that the binding of LIN-1 to the *egl-1* promoter is important for the cell-killing activity of *egl-1*.

To test whether this LIN-1-mediated cell-killing activity of *egl-1* may depend on LIN-3 signaling, we examined whether a *lin-3(lf)* mutation may reduce the rescuing activity of the mutant *egl-1* transgene by scoring surviving ABpl/rpppapp (Figure 6C) and embryonic cell corpses (Figure 6D). As shown in Figure 6C and 6D, the *lin-3(lf)* mutation reduced the rescuing activity of the wild-type *egl-1* transgene, but failed to reduce the rescuing activity of the mutant *egl-1* transgene. These results show that binding of LIN-1 to the *egl-1* promoter is critical for LIN-3 signaling to promote PCD. These data support the idea that LIN-3 acts through LIN-1 to promote PCD by transcriptional activation of *egl-1*.

## Discussion

### LIN-3 signaling promotes PCD through LET-60-MPK-1 signaling and LIN-1 to activate *egl-1* transcription

LIN-3-induced LET-60-MPK-1 signaling is important in multiple aspects of development in *C. elegans* [39]–[45]. In this study, we provide evidence that it is also essential for specific somatic PCD. Mutants defective in genes coding for proteins in this pathway showed reduced PCD in specific cells, ABpl/rpppapp, g1A sisters, and I1 sisters. In contrast, over-activation of this pathway resulted in ectopic deaths of specific cells, such as gland cells. These results show that LIN-3-induced LET-60-MPK-1 signaling is crucial for the death-versus-life fate of specific cells. In addition, the downstream transcription factor LIN-1, but not LIN-31, was shown to act in the LIN-3-induced LET-60-MPK-1 signaling pathway to regulate PCD via the target gene *egl-1*. Furthermore, we showed that LIN-1 bound directly to the *egl-1* promoter *in vitro* and that the LIN-1 binding site of the *egl-1* promoter was important for the cell death-promoting function of LIN-3 *in vivo*.

Out of 35 embryonic PCDs we analyzed, including the first 13 PCDs in the AB lineage and 22 pharyngeal PCDs, the *lin-3*-to-*lin-1* signaling pathway is important for the death of ABpl/rpppapp, g1A sisters, and I1 sisters. ABpl/rpppapp and I1 sisters undergo PCD before the comma stage, and g1A sisters die during the comma stage [6]. Our embryonic cell corpse data revealed that mutants defective in the *lin-3*-to-*lin-1* signaling pathway had less cell corpses at not only the comma stage, but also 1.5-fold and 2-fold stages. However, the identities of dying cells affected at the latter two stages have not yet been determined, partly because no cell-specific markers are available to test for their survival in the mutants. It is possible that those lineages where PCDs might be more severely affected have not yet been analyzed in our work and, if so, the *lin-3* signaling pathway might play a predominant role in these PCDs as compared to the death of ABpl/rpppapp, g1A sisters, and I1 sisters. Since the mutants defective in the *lin-3* signaling pathway showed a partially penetrant phenotype in the survival of ABpl/rpppapp, g1A sisters, and I1 sisters, other genes or pathways, in addition to the *lin-3* signaling pathway, are required to efficiently promote the death of, at least, ABpl/rpppapp, g1A sisters, and I1 sisters.

Although we have not yet characterized all affected embryonic PCDs, the data obtained using either the specific cell death assays or the embryonic cell corpse counting assays were similar. For example, the *lin-3*, *let-23*, *let-60*, *mpk-1* and *lin-1* single mutants had the same phenotype as their respective single mutants in the *lin-3* or *let-23* mutant background or feeding *lin-3* or *let-23* RNAi (Figure 1A, 1D, 1G, 5A, and Table S5). Overexpression of *lin-3* using its endogenous promoter or misexpression of *lin-3* in the intestine resulted in ectopic gland cells and increased numbers of embryonic cell corpses during comma to two-fold stages. Both phenotypes could be suppressed by loss of *let-23* or components of the core PCD pathway (Figure 2 and Table S2). In addition, the wild-type *egl-1* genomic DNA fully rescued the *egl-1* cell death defect, but the mutant *egl-1* genomic DNA with a mutation in the LIN-1 binding site could only rescue the *egl-1* cell death defect to a similar extent as the *lin-3* mutant (Figure 6C and 6D). Furthermore, loss of *lin-3* did not aggravate the PCD defect of the *egl-1; Ex[egl-1(mut)]* transgenic animals (Figure 6C and 6D). Therefore, the *lin-3*-to-*lin-1* signaling pathway may promote the death of ABpl/rpppapp and g1A sisters as well as other yet uncharacterized doomed cells by the same molecular mechanism. However, we cannot rule out the possibility that *let-23*, *mpk-1*, *let-60* or *lin-1* may affect other aspects of PCD, rather than promoting the PCD fate, in the yet unidentified doomed cells.

In the genetic interaction experiments, the *let-23(n1045)* mutation does not completely eliminate the cell death-promoting function of *syIs1*: the mutation reduced the numbers of ectopic embryonic cell deaths (Table S2) to the wild-type level, but not to the *let-23(n1045)* mutant level. The *let-23(n1045)* allele may still have a residual *let-23* activity [52] and therefore cannot fully block the *lin-3* signaling from *syIs1*. Alternatively, *syIs1* may exert its PCD-promoting function through both LET-23-dependent and LET-23 independent pathways to cause ectopic cell deaths.

Previous studies showed that LIN-1 is negatively regulated by the upstream LIN-3-induced LET-60-MPK-1 signaling pathway during vulval induction [46]. We found that the LIN-1 function is positively regulated by this signaling pathway to promote the PCD fate of specific cells. During vulval development, although *lin-1* inhibits most target gene expression downstream of the LET-60-MPK-1 pathway, *lin-1* is also required positively for the expression of several downstream genes, such as *egl-17* [66]. Our data indicated that *lin-1* activates *egl-1* expression in promoting PCD of specific cells. Interestingly, both of these *lin-1*-mediated gene expression processes are positively regulated by the LET-60-MPK-1 pathway and do not require *lin-31*. It appears that LIN-1 could have either a positive or negative function on gene expression, depending on the cellular context and target genes.

LIN-3 signaling was also found to activate *ced-4* transcription, but *egl-1* transcription probably accounted for most of the LIN-3 signaling-mediated cell death, as, in the *egl-1* mutant, the mutant *egl-1* transgene lacking the LIN-1 binding site could only restore the penetrance of ABpl/rpppapp death and the embryonic cell corpse number to the levels seen in the *lin-3* mutant.

A previous study showed that dying cells that inappropriately survive generally adopt the fate of the sister or lineal homolog if the latter cells are terminally differentiated [11]. We observed that the g1A sister, when survived in the *lin-3* or *ced-3* mutant, expressed the gland cell marker *P_B0280.7_::4Xnls::mrfp*, indicating that the g1A sister adopted its sister cell fate. To rule out the possibility that the *lin-3* mutation may cause cell fate transformation and hence block cell death, we examined surviving ABpl/rpppapp in detail in the *lin-3* mutant. ABpl/rpppapp was chosen because the sister of ABpl/rpppapp divides once and generates a hypodermal cell and a phasmid sheath cell [6]. It has been shown that dying cells that inappropriately survive do not adopt their sister cell fates, if the latter cells undergo cell division and the resulting descendent cells then differentiate [11]. The observation that surviving ABpl/rpppapp in the *lin-3* or *egl-1* mutant did not express the hypodermal cell-specific genes *dpy-7* and *ajm-1*, as assayed by their respective reporters *P_dpy-7_::yfp* and *ajm::gfp*, suggesting that surviving ABpl/rpppapp did not further divide or differentiate to generate a hypodermal cell. This result argues against the possibility that the *lin-3* mutation affects ABpl/rpppapp survival indirectly through cell fate transformation to its sister or niece cell.

### An appropriate level of extrinsic LIN-3 signaling is important for the precise fine-tuning of the life-versus-death fate of cells

Our data support the following model explaining how different levels of LIN-3 expression or activity might regulate PCD (Figure 6E). In the wild-type, after binding LIN-3, LET-23 transduces the cell death-promoting signal through the LET-60-MPK-1 pathway, leading to LIN-1 activation, followed by *egl-1* transcription, and the demise of specific cells. However, mutations in either the LIN-3-induced LET-60-MPK-1 signaling pathway or LIN-1 or a defect in the LIN-1-binding site of the *egl-1* promoter reduces *egl-1* transcription and thus the probability that a cell undergoes PCD. In contrast, overexpression of *lin-3* enhances *egl-1* transcription and leads to ectopic cell deaths. Therefore, LIN-3 signaling may act to specify the death of specific cells and fine-tunes the life-versus-death fate of these cells by modulating transcription of *egl-1*.

Although EGF has long been known to function in the process of cell proliferation and survival, some reports showed that it also promotes apoptosis [30]–[32]. In some cases, the difference in cellular outcome appears to depend on its concentration, with a low EGF concentration promoting cell proliferation and a high concentration inducing apoptosis [67], [68]. Cells expressing different levels of EGFR also show different responses to the same amount of EGF, as it has been reported that exogenous EGF attenuates cell death in rats, which express low levels of EGFR, but promotes cell death in mice, which express high levels of EGFR [69]. Thus, it is likely that overactivation of EGF signaling triggers apoptosis. This may be a conserved physiological mechanism for preventing unrestricted proliferation when cells respond to persistent or hyperactivated EGF signaling. In *C. elegans*, cells may use this strategy to fine-tune PCD: cells that are doomed to die may express a high level of LET-23 or its downstream signaling components. This may either avoid inappropriate survival of cells that occasionally escape from the cell death machinery or preset a cell in a sensitized stage to respond quickly when it receives the death signal. This may also explain why some cells are more sensitive to apoptotic stimuli than others.

Our data show that different cells appear to show different susceptibility to *lin-3* signaling in PCD at, at least, three different levels. First, ABpl/rpppapp, g1A sisters, and I1 sisters require *lin-3* signaling for PCD and are sensitive to the wild-type level of *lin-3* signaling. Second, g1 and g2 cells are sensitive to a high level of *lin-3* signaling in PCD, since they survive in the wild-type but can be induced to undergo PCD by the *syIs1* transgene. Third, cells, such as PLM, survive even in the presence of the transgene *syIs1* and are therefore insensitive to *lin-3* signaling for their life vs death fate. These results together show a complexity of extrinsic signaling in PCD regulation.

### The LET-60-MPK-1 pathway promotes germline and developmental cell deaths

Consistent with our data, mutants defective in *ksr-1*, a positive regulator of the RAS signaling pathway, have reduced embryonic cell corpse numbers [70]. The LET-60-MPK-1- and PI3K-mediated signaling pathways are also known to be involved in physiological or genotoxic stress-induced germ cell death in *C. elegans* [48]–[51], although the ligand and receptor for these pathways have not yet been identified. The LET-60-MPK-1 pathway regulates the apoptotic process of irradiation-induced germ cell death by restricting CEP-1 protein expression to cells in late pachytene. More importantly, MPK-1, the *C. elegans* ERK, is activated following irradiation and is required for *egl-1* transcription in irradiation-induced germ cell death [50]. This result and our present findings show that the LET-60-MPK-1 pathway regulates *egl-1* transcription to promote both physiological somatic PCD and genotoxic stress-induced germline cell death. In mammalian cells, activation of the RAS-ERK pathway upregulates Bcl-X_L_ transcription [26]. It has also been shown that EGF regulates the expression of Mcl-1, a member of the anti-apoptotic Bcl-2 family, through activation of MAPK signaling and the direct binding of Elk-1, a member of the ETS gene family, to the Mcl-1 promoter [71]. Thus, the interaction of the RAS-ERK pathway and Bcl-2 family members, such as *egl-1*, Bcl-X_L_, and Mcl-1_,_ may be a conserved strategy for regulating cell death or survival in evolution.

### Pathological and physiological roles of EGFR in promoting cell death

It has been reported that cisplatin, a widely used chemotherapeutic agent for human cancers, causes nephrotoxicity in patients and induces extensive death of the proximal tubules in mice or cultured proximal tubule cells [72]–[74]. Cisplatin activates EGFR and ERK to trigger apoptosis in cultured proximal tubule cells [74]. In addition, in the autoimmune cutaneous disease pemphigus vulgaris, the body produces autoantibodies that can induce EGFR activation and ERK phosphorylation, leading to activation of caspase-3 [75]. EGFR activation-induced RAS signaling has also been implicated in age-related brain degeneration in *Drosophila* [76]. Taken together, these results and our data show that EGFR activation-induced cell death is a conversed phenomenon and plays an important role in pathological and physiological conditions.

Mutations that cause constitutive activation of EGFR are commonly observed in human cancers. Specific antibodies or inhibitors against activated EGFR have been developed and used as targeted therapeutics [77]. However, previous studies in human cell lines [30]–[32] and our present results show that overactivation of EGF signaling can promote apoptosis, raising the concern that drugs that antagonize EGFR signaling might have an anti-apoptotic effect and thus a potential tumor-promoting side-effect, depending on the cellular context. Further information on the molecular basis of the distinct divergent cellular responses of cell proliferation, cell survival, and death to EGF signaling is important for our understanding of the physiological roles of EGF and EGFR signaling during development and homeostasis and for providing perspectives for optimizing the strategy of anticancer drug design.

## Materials and Methods

### General methods and strains

Unless otherwise stated, *C. elegans* strains were maintained at 20°C as described previously [78]. The N2 Bristol strain was used as the wild-type strain. Alleles used:

Linkage group (LG)I: *aap-1(m889)*, *aap-1(ok282)*, *age-2(yw23)*, *daf-16(m26)*, and *mek-2(ku114)*.

LGII: *age-1(hx546)*, *age-1(m333)*, *let-23(n1045)*, *let-23(sy1)*, *let-23(sy97)*, *lin-31(gk569)*, *lin-31(n301)*, *lin-31(n1053)*, *plc-3(tm1340)*, and *rrf-3(pk1426)*.

LGIII: *ced-4(n1162)*, *ced-9(n1950)*, *mpk-1(ku1)*, *mpk-1(n2521)*, *unc-32(e189)*, *unc-79(e1068)*, and *syIs107[P_lin-3_::pes-10::gfp]*.

LGIV: *ced-3(n717)*, *let-60(n1046)*, *let-60(sy93)*, *lin-1(n1761)*, *lin-1(n303)*, *lin-1(n1047)*, *lin-1(sy254)*, *lin-3(e1417)*, *lin-3(n378)*, *lin-45(n2506)*, and *plc-4(ok1215)*.

LGV: *egl-1(n1084n3082)*, *plc-2(ok1761)*, *sos-1(cs41)*, and *bcIs37[P_egl-1_::his-24::gfp]*.

LGX: *plc-1(rx1)*, *sem-5(n1779)*, and *syIs1[lin-3(+)]*.

All alleles are described in WormBase (http://www.wormbase.org/). *tpIs8* is an integrated version of *tpEx270[P_B0280.7_::4Xnls::mrfp; unc-119(+)]* (this report). *P_B0280.7_::4Xnls::mrfp* is expressed in g1 and g2 gland cells [55].

### RNA interference (RNAi)

There are about 40 receptor tyrosin kinase (rtk) genes in the *C. elegans* genome. To test the involvement of these genes in promoting PCD, RNAi of the individual rtk genes was performed by feeding [79], and the cell corpses of treated embryos were scored. In this screen, only *let-23* RNAi significantly decreased cell corpse numbers at the comma and 1.5-fold stages. To characterize the effect of the *lin-3* signaling pathway on the number of embryonic cell corpses, the *rrf-3(pk1426)* mutant, which is sensitive to RNAi [80], was treated by feeding RNAi at 20°C, as previously described [81]. In other RNAi experiments, animals were treated with RNAi without the *rrf-3(pk1426)* mutation. The *lin-3* or *let-23* RNAi treatment caused more than 50% rod-like larval lethality both in the wild-type and *rrf-3(pk1426)* mutant, confirming that *lin-3* and *let-23* RNAi works well in these experiments. RNAi plasmids were obtained from the J. Ahringer RNAi library. L4 hermaphrodites were put onto the RNAi plates, and F1 were scored for the number of embryonic cell corpses, surviving ABpl/rpppapp, or extra gland cells. The empty vector L4440 was used as negative control.

### Transgenic animals

Germline transformation experiments were performed as described previously [82]. To observe the expression pattern of *let-23*, *P_let-23_::4Xnls::gfp* (50 ng/µl) was injected into *unc-119(ed3)* worms with the coinjection markers *unc-119* [83] (50 ng/µl) and *P_ced-1_::1Xnls::mrfp* (50 ng/µl). To examine the expression pattern of *lin-3* and *let-23*, *P_let-23_::4Xnls::mrfp* (200 ng/µl) was injected into an integrated transgene *syIs107[P_lin-3_::pes-10::gfp]* with the coinjection markers *P_elt-2_::gfp* (50 ng/µl) [61]. To test whether secreted LIN-3 rescued the cell death defect of the *lin-3* mutant and caused ectopic cell deaths, *P_elt-2_::gfp* or *P_elt-2_::lin-3::gfp* (50 ng/µl) was injected into the *lin-3(e1417)* mutant and wild-type with the coinjection marker *sur-5::gfp* (50 ng/µl). To test the effect of LIN-1 overexpression on PCD, plasmids pPD49.78 and pPD49.83 (containing *P_hsp_* without an insert) or *P_hsp_::lin-1* (50 ng/µl) was co-injected with the coinjection marker *sur-5::gfp* (50 ng/µl) into animals carrying the integrated transgene *tpIs8*. To examine the importance of the LIN-1 binding site in the *egl-1* promoter, wild-type (pBC08) [7] or mutant *egl-1* (2 ng/µl) was injected into either *egl-1(n1084n3082)* or *lin-3(e1417); egl-1(n1084n3082)* mutants with the coinjection marker *sur-5::gfp* (50 ng/µl).

### Heat shock treatment

Embryos were incubated at 20°C or heat shocked at 33°C for 10–60 min and then moved to 20°C. The number of g1 and g2 gland cells was scored at the L4 stage using the *tpIs8* transgene after three days.

### Molecular biology

Standard methods of cloning, sequencing, and PCR amplification were used. To generate *P_let-23_::4xnls::gfp*, the *let-23* promoter was amplified by PCR using primers 5′-gtctagagcatctgcacttggg-3′ and 5′-gggatccgcctcccag-3′ and the resulting PCR fragment was inserted into the pPD122.56 vector to generate pYW1242. Full-length *lin-3* cDNA was obtained by RT-PCR using worm total RNA and the primers 5′-CCACCGGTATGCGGAAAATGCTAC-3′ and 5′-CCACCGGTTTTGTGTGTCGAATCATTGG-3′. To obtain *P_elt-2_::lin-3::gfp*, the *elt-2* promoter, previously amplified by PCR using 5′-TTGGATCCCGGTGAAACTCTCTTGG-3′ and 5′-CCGGATCCCAGTGGCACCTAAAACATC-3′, was cloned into the BamHI site of pPD95.75 and *lin-3* cDNA was cloned into the AgeI site to generate pYW1243. To generate *P_hsp_::lin-1*, a full-length *lin-1* cDNA was amplified from the yk1150h12 plasmid by PCR using 5′-ccggatccatgaatcacattgaccttttg-3′ and 5′-ttccatggctacaaagttggcatttttatg-3′, and the resulting PCR fragment was inserted into the heat-shock vectors pPD49.78 and pPD49.83 to generate pYW1245 and pYW1246, respectively. The DNA fragment corresponding to the LIN-1 DNA binding domain was amplified by PCR using the yk1150h12 plasmid as template and 5′-CCGGATCCATGAATCACATTGACCTTTTG-3′ and 5′-TTGGATCCTTTCGTTGGCGGCTGCGG-3′ as primers [65], then the PCR product was inserted into vector pGEX-4T-1 to generate plasmid pYW1247. The pBC08 plasmid contains the *egl-1* genomic fragment, which rescues the cell death defect of *egl-1* mutants [7]. To generate mutant *egl-1* with a defect in the LIN-1 binding site, two partial fragments of *egl-1* from pBC08 were first amplified by PCR using primer pairs 5′-caatttatagtaaagttaacttca-3′ plus 5′-cacatacagtagcttgaattcaactatacatctg-3′ and 5′-aattcaagctactgtatgtgtcacttccaccacc-3′ plus 5′-ggccgctctagaactagtgg-3′. The products were then used as templates for fusion PCR to generate a larger fragment to replace the corresponding region in pBC08 to generate pYW1248. This mutant *egl-1* was identical to wild-type *egl-1* except that the core LIN-1 binding motif was changed from CAGGAA to GCTACT.

### Cell death assays

Cell corpses were counted at the indicated developmental stages and the number of extra cells in the anterior and posterior pharynx of L4 hermaphrodites was scored using Nomarski optics as described previously [54]. To analyze the first 13 cell deaths that occur in the AB lineage during embryogenesis, embryos were mounted onto 4% agar pads and recorded using a four-dimensional DIC microscopy analysis as described previously [84]. The survival of ABpl/rpppapp was examined by its localization in the tail with the aid of *sur-5::gfp*. To count the number of gland cells in the pharynx, the integrated transgene *tpIs8[P_B0280.7_::4Xnls::mrfp]* was used as a marker for these gland cells [55], and the number of the mRFP-positive cells was scored at L4 stage by fluorescence microscopy.

### Quantitative real-time reverse transcriptase (RT)-PCR

Total RNA was isolated from embryos developing up to the comma stage and purified by chloroform extraction and isopropanol precipitation, then 500 ng was reverse transcribed into cDNA using RevertAid H Minus First Strand cDNA Synthesis Kits (Fermentas). Equal amounts of the different cDNAs were used as the template for gene-specific PCR with appropriate primers. Water was used as negative control and *tbg-1* RT-PCR was performed as an internal control. The CT values of the test genes were normalized to that of *tbg-1* and relative expression levels were derived using the comparative CT method [85].

### Quantification of *P_egl-1_::gfp* intensity in ABprpppapp

To measure the *P_egl-1_::gfp* expression within ABprpppapp, fluorescence micrographs were captured using a Zeiss AxioImager M2 microscope (Zeiss) equipped with a charge-coupled device camera. GFP intensity was determined using Image J by measuring the fluorescence signals within ABprpppapp (Ia) and its neighboring region (Ib) of *ced-3(717)* and *lin-3(e1417) ced-3(n717)* mutants. As a control, the fluorescence signals within two cells near ABprpppapp (Ia) and its neighboring region (Ib) of *ced-3(717)* and *lin-3(e1417) ced-3(n717)* mutants was determined. The value of Ia minus the value of Ib was used as the value for relative GFP intensity, and the relative GFP intensity of the *ced-3(n717)* mutant was used for normalization.

### Prediction of potential LIN-1 binding sites

The prediction of potential LIN-1 binding sites was performed at http://www.cbrc.jp/research/db/TFSEARCH.html by submitting the sequence from +174 to +5820 (5′-3′) downstream of the stop codon of the *egl-1* gene and the sequence from −1914 to −837 (5′-3′) upstream of the stop codon [62]. Four binding sites for Elk-1, the mammal LIN-1 ortholog, were predicted; these were AGCTTTCCGGCTCA (P1), CGATCCGGAAATCC (P2), TAGTTGAATTCAACAGGAAGTATGTGTCACTTCC (P3), and CAATTTCCGGTAAT (P4). P2 and P3, but not P1 or P4, are conserved in *C. briggsae*. P2 has been shown to be important for the PCD of the neurosecretory-motor (NSM) neuron [19], but *lin-3* and *let-23* do not seem to be involved in the death of NSM sister cell (Figure 1D), we therefore focused on P3 in this study.

### Electrophoretic mobility shift assay (EMSA)

Expression of GST and GST::LIN-1(DBD) was induced in *Escherichia coli* strain BL21 carrying the respective plasmids pGEX-4T-1 and pYW1247 by addition of 0.4 mm isopropyl thiogalactoside and incubation for 1 h at 37°C. The cells were then harvested and disrupted by sonication in phosphate-buffered saline containing protease inhibitor cocktail (Calbiochem) and 1% Triton X-100, and the proteins were purified on glutathione Sepharose (Amersham Biosciences) according to the manufacturer's protocol. The amount of protein was measured by the Bradford method (Biorad). The sense and antisense oligonucleotides containing the predicted LIN-1 binding sequences, which were synthesized and biotinylated at the 3′ end (Genomics), were mixed in annealing buffer (5 mM NaCl, 1 mM Tris-HCl, 1 mM MgCl_2_, and 0.1 mM dithiothreitol, pH 7.9) at a final concentration of 1 µM, denatured at 98°C for 2 min, and allowed to anneal by slowly cooling the mixture to 25°C. Unlabeled probes were used as competitors in competition experiments. The sequences of the probes were: wild-type: 5′-gaattcaacaggaagtatgtgtcacttccaccac-3′ and 5′-gtggtggaagtgacacatacttcctgttgaattc-3′; mutant: 5′- gaattcaagctactgtatgtgtcacttccaccac-3′ and 5′-gtggtggaagtgacacatacagtagcttgaattc-3′. EMSA were performed using LightShift Chemiluminescent EMSA kits (Pierce), following the manufacturer's instruction. The mixture was separated at 100 V at 4°C on a 5.5% non-denaturing polyacrylamide gel, then the DNA was transferred to a charged nylon membrane (Millipore) at 150 mA for 30 min and cross-linked with UV light using the auto crosslink option on a UV Stratalinker 1800 (Stratagene). The membrane was then blocked, washed, and exposed to X-ray film following the manufacturer's protocol.

### Statistical analysis

In histograms, the values are shown as the mean and standard deviation (SD). Data derived from different genetic backgrounds at a specific developmental stage were compared using Student's two-tailed unpaired *t*-test. Data were considered statistically different at *P*<0.05. Fisher's exact test was used when comparing the percentages of surviving or missing cells in different genetic backgrounds. Data were considered statistically different at *P*<0.05.

## Supporting Information

Figure S1 **Loss of *lin-3* causes the disappearance of ABpl/rpppapp corpse(s).** The first 13 cells that die in the AB lineage during embryogenesis were examined by four-dimensional DIC microscopy in thirteen *lin-3(e1417)* mutant embryos as described in Materials and Methods. (A) The ratio of embryos that did not have the indicated cell corpse was shown. (B) A representative DIC image of a *lin-3* (*(e1417)* embryo showing apoptotic ABpl/rpppapp (indicated by arrow heads). (C) A representative DIC image of a *lin-3* (*(e1417)* embryo showing healthy ABprpppapp (indicated by open arrow head) and apoptotic ABplpppapp (indicated by arrow head).
(TIF)

Figure S2 **The *lin-3(e1417)* mutation does not affect the duration of the first 13 cell corpses derived from the AB lineage.** The duration of cell corpses in the wild-type (white bars) and *lin-3(e1417)* mutant (gray bars) embryos was measured by a four-dimensional DIC microscopy analysis. More than 13 embryos were analyzed for each genotype. The y axis represents the percentage of cell corpses in a specific duration range (shown on the x axis). Numbers in parentheses indicate the average duration time of cell corpses (mean ± SD) for each genotype. There is no significant difference between the wild-type and *lin-3(e1417)* mutant embryos (*P* = 0.24, two-tailed *t* test).
(TIF)

Table S1 **Analysis of extra surviving cells in the pharyngeal region in the *ced-3(n2427)* sensitized background.**
(PDF)

Table S2 ***lin-3* requires *let-23* and the core PCD pathway to increase numbers of embryonic cell corpses.**
(PDF)

Table S3 **Mutants defective in the LET-60-MPK-1 pathway have reduced numbers of cell corpses.**
(PDF)

Table S4 **The PI3K pathway and PLC genes are not involved in embryonic PCD.**
(PDF)

Table S5 **The *lin-1* mutants, but not *lin-31* mutants, have reduced numbers of cell corpses.**
(PDF)
