# Supplementary figures and images for: LIN-3/EGF Promotes the Programmed Cell Death of Specific Cells in Caenorhabditis elegans by Transcriptional Activation of the Pro-apoptotic Gene egl-1
Source: PLoS Genet. 2014 Aug 21;10(8):e1004513. doi: 10.1371/journal.pgen.1004513 (PMC4140636; doi:10.1371/journal.pgen.1004513)

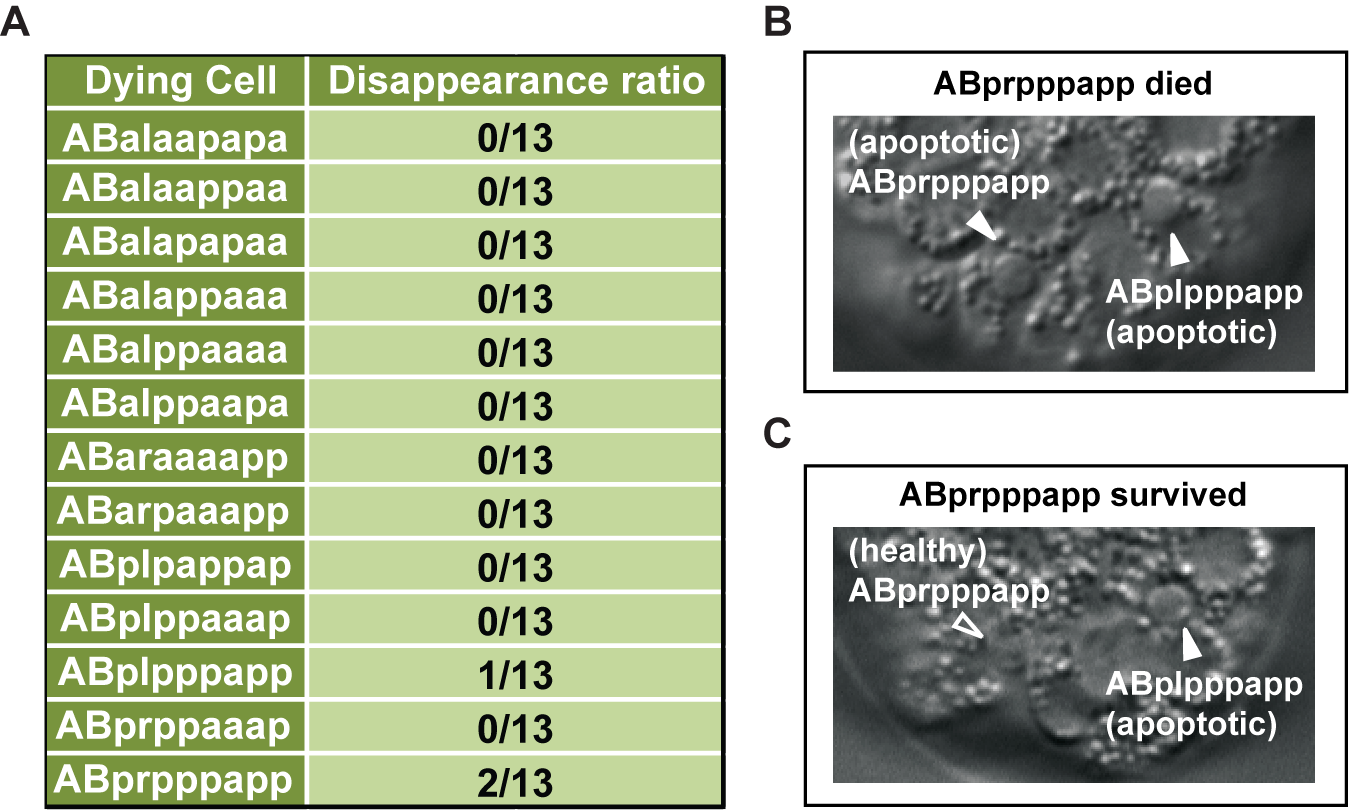

Supplement: Figure S1 — Loss of lin-3 causes the disappearance of ABpl/rpppapp corpse(s). The first 13 cells that die in the AB lineage during embryogenesis were examined by four-dimensional DIC microscopy in thirteen lin-3(e1417) mutant embryos as described in Materials and Methods. (A) The ratio of embryos that did not have the indicated cell corpse was shown. (B) A representative DIC image of a lin-3 ((e1417) embryo showing apoptotic ABpl/rpppapp (indicated by arrow heads). (C) A representative DIC image of a lin-3 ((e1417) embryo showing healthy ABprpppapp (indicated by open arrow head) and apoptotic ABplpppapp (indicated by arrow head). (TIF) [file pgen.1004513.s001.tif]

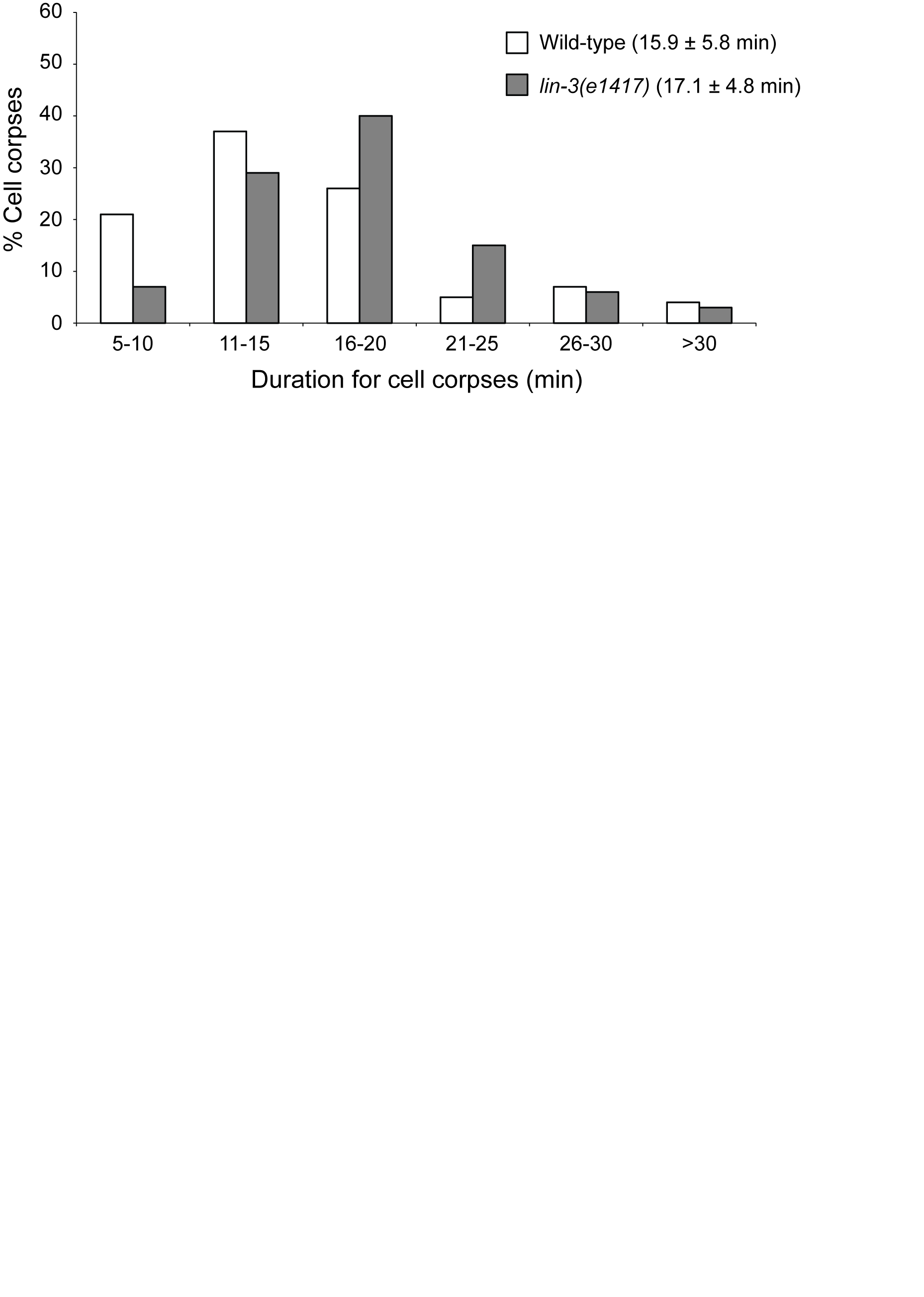

Supplement: Figure S2 — The lin-3(e1417) mutation does not affect the duration of the first 13 cell corpses derived from the AB lineage. The duration of cell corpses in the wild-type (white bars) and lin-3(e1417) mutant (gray bars) embryos was measured by a four-dimensional DIC microscopy analysis. More than 13 embryos were analyzed for each genotype. The y axis represents the percentage of cell corpses in a specific duration range (shown on the x axis). Numbers in parentheses indicate the average duration time of cell corpses (mean ± SD) for each genotype. There is no significant difference between the wild-type and lin-3(e1417) mutant embryos (P = 0.24, two-tailed t test). (TIF) [file pgen.1004513.s002.tif]
