# Supplementary material for: LIN-3/EGF Promotes the Programmed Cell Death of Specific Cells in Caenorhabditis elegans by Transcriptional Activation of the Pro-apoptotic Gene egl-1
Source: PLoS Genet. 2014 Aug 21;10(8):e1004513. doi: 10.1371/journal.pgen.1004513 (PMC4140636; doi:10.1371/journal.pgen.1004513)
Supplement: Table S1 — Analysis of extra surviving cells in the pharyngeal region in the ced-3(n2427) sensitized background. (PDF) [file pgen.1004513.s003.pdf]

Table S1. Analysis of extra surviving cells in the pharyngeal region in the *ced-3(n2427)* sensitized background.

Genotype	% of extra surviving cells in the pharyngeal region											
	% g1 sister	% I1 sister	% M1 sister	% m7 sister	% M4 sister	% g2 sister	% m1 sister	% MC sister	% I2 aunt/sister	% e1 aunt/sister	% m2 sister	% NSM sister
ced-3	10	10	30	15	5	55	20	15	10	0	15	0
lin-3 ced-3	35	40	60	35	15	70	35	35	10	0	20	5
let-23; lin-3	40	40	70	20	10	70	40	20	15	5	10	0

Extra surviving cells were counted in the anterior and posterior pharynx of L4 hermaphrodites using Nomarski optics (n=20).

Alleles used: *lin-3(e1417)* , *let-23(n1045)* , and *ced-3(n717)* .
