# Supplementary material for: LIN-3/EGF Promotes the Programmed Cell Death of Specific Cells in Caenorhabditis elegans by Transcriptional Activation of the Pro-apoptotic Gene egl-1
Source: PLoS Genet. 2014 Aug 21;10(8):e1004513. doi: 10.1371/journal.pgen.1004513 (PMC4140636; doi:10.1371/journal.pgen.1004513)
Supplement: Table S2 — lin-3 requires let-23 and the core PCD pathway to increase numbers of embryonic cell corpses. (PDF) [file pgen.1004513.s004.pdf]

Table S2. *lin-3* requires *let-23* and the core PCD pathway to increase numbers of embryonic cell corpses.

	Number of embryonic cell corpses[a]				
**Genotype**	**Comma**	**1.5-fold**	**2-fold**	**3-fold**	**4-fold**
Wild-type	10.0 ± 1.6	11.4 ± 1.5	10.0 ± 1.9	2.1 ± 1.7	0.5 ± 0.7
syIs1	12.4 ± 2.4**	13.2 ± 2.2**	13.1 ± 2.1**	2.5 ± 1.8	0.5 ± 0.6
let-23(n1045); syIs1	9.9 ± 2.4††	10.8 ± 1.9††	9.3 ± 2.6††	2.2 ± 1.5	0.6 ± 0.6
egl-1(n1084n3082)	0.1 ± 0.3	0.1 ± 0.3	0.1 ± 0.3	0.0 ± 0.0	0.0 ± 0.2
egl-1(n1084n3082); syIs1	0.1 ± 0.3	0.0 ± 0.0	0.0 ± 0.0	0.0 ± 0.0	0.0 ± 0.2
ced-9(n1950)	0.1 ± 0.3	0.4 ± 0.5	1.2 ± 1.1	0.5 ± 0.6	0.4 ± 0.6
ced-9(n1950); syIs1	0.3 ± 0.5	0.4 ± 0.6	0.8 ± 0.8	0.7 ± 0.7	0.7 ± 0.7
ced-4(n1162)	0.1 ± 0.3	0.1 ± 0.3	0.0 ± 0.0	0.0 ± 0.0	0.0 ± 0.0
ced-4(n1162); syIs1	0.1 ± 0.3	0.1 ± 0.3	0.0 ± 0.0	0.0 ± 0.0	0.0 ± 0.0
ced-3(n717)	0.1 ± 0.3	0.1 ± 0.3	0.1 ± 0.3	0.0 ± 0.0	0.0 ± 0.0
ced-3(n717); syIs1	0.1 ± 0.3	0.1 ± 0.3	0.1 ± 0.3	0.0 ± 0.0	0.0 ± 0.0

[a]Cell corpses scored at the indicated developmental stages are presented as the mean ± SD. (n≥30). *syIs1* is a chromosomal integrant of a repetitive *lin-3* transgene. *syIs1* was compared to wild-type (*$P<0.05$ and **$P<0.001$, two-tailed *t* test) and *let-23(n1045); syIs1[lin-3(+)]* were compared to *syIs1[lin-3(+)]* (†$P<0.05$ and ††$P<0.001$, two-tailed *t* test).
