# Supplementary material for: LIN-3/EGF Promotes the Programmed Cell Death of Specific Cells in Caenorhabditis elegans by Transcriptional Activation of the Pro-apoptotic Gene egl-1
Source: PLoS Genet. 2014 Aug 21;10(8):e1004513. doi: 10.1371/journal.pgen.1004513 (PMC4140636; doi:10.1371/journal.pgen.1004513)
Supplement: Table S3 — Mutants defective in the LET-60-MPK-1 pathway have reduced numbers of cell corpses. (PDF) [file pgen.1004513.s005.pdf]

Table S3. Mutants defective in the LET-60-MPK-1 pathway have reduced numbers of cell corpses.

Genotype	Number of cell corpses[a]				
	Comma	1.5-fold	2-fold	3-fold	4-fold
Wild-type (at 20℃)	10.0 ± 1.6	11.4 ± 1.5	10.0 ± 1.9	2.1 ± 1.7	0.5 ± 0.7
Wild-type (at 25℃)	10.1 ± 2.5	11.1 ± 2.1	10.0 ± 1.9	2.2 ± 1.9	0.5 ± 0.7
let-60(n1046gf)	9.5 ± 2.3	10.7 ± 3.1	9.7 ± 2.4	2.0 ± 1.7	0.6 ± 0.7
unc-32(e189)	9.7 ± 1.6	10.9 ± 1.8	9.8 ± 2.0	1.7 ± 1.5	0.5 ± 0.7
unc-79(e1068)	9.3 ± 2.2	11.3 ± 1.7	10.0 ± 2.1	2.5 ± 1.5	0.5 ± 0.7
sem-5(n1779)	7.6 ± 1.6**	9.5 ± 1.5**	6.3 ± 2.3**	1.5 ± 1.4	0.3 ± 0.5
sos-1(cs41) (at 25℃)[b]	7.7 ± 2.7**	10.9 ± 3.5	8.9 ± 2.8	2.0 ± 1.7	0.3 ± 0.5
let-60(sy93)	6.5 ± 3.0**	8.5 ± 2.4**	6.2 ± 3.3**	2.3 ± 1.7	0.4 ± 0.7
lin-45(n2506)[c]	6.7 ± 2.6**	8.2 ± 2.0**	6.3 ± 3.4**	1.5 ± 1.6	0.4 ± 0.6
mek-2(ku114)[c]	7.1 ± 2.6**	7.9 ± 2.6**	5.6 ± 3.0**	1.7 ± 1.6	0.5 ± 0.8
mpk-1(ku1)[d]	7.7 ± 2.5**	9.0 ± 1.1**	6.5 ± 2.5**	2.2 ± 1.7	0.4 ± 0.6
mpk-1(n2521)[e]	6.9 ± 2.3**	8.0 ± 2.4**	5.9 ± 2.2**	1.9 ± 1.5	0.5 ± 0.7
mpk-1(n2521)[c]	7.3 ± 2.3**	8.4 ± 2.0**	7.0 ± 3.5**	2.4 ± 2.0	0.5 ± 0.9
Control RNAi	9.2 ± 2.9	10.2 ± 3.7	9.7 ± 1.8	2.1 ± 1.8	0.5 ± 0.7
let-60 RNAi	7.3 ± 1.8*	8.5 ± 2.2*	6.4 ± 3.0**	1.8 ± 2.0	0.4 ± 0.6

[a]Cell corpses scored at the indicated developmental stages are presented as the mean ± SD. (n≥30). The *rrf-3(pk1426)* mutant was used to enhance the RNAi effect in RNAi experiments. Mutants embryos were compared to wild-type, and *let-60* RNAi treatment was compared to control RNAi treatment (**P*<0.05 and ***P*<0.001, two-tailed *t* test). [b]*sos-1(cs41)* is temperature-sensitive and its cell corpse profile was scored at 25℃. [c]Mutants also contain *let-60(n1046gf)*. [d]Mutants also contain *unc-32(e189)*. [e]Mutants also contain *unc-79(e1068).*
