# Supplementary material for: LIN-3/EGF Promotes the Programmed Cell Death of Specific Cells in Caenorhabditis elegans by Transcriptional Activation of the Pro-apoptotic Gene egl-1
Source: PLoS Genet. 2014 Aug 21;10(8):e1004513. doi: 10.1371/journal.pgen.1004513 (PMC4140636; doi:10.1371/journal.pgen.1004513)
Supplement: Table S4 — The PI3K pathway and PLC genes are not involved in embryonic PCD. (PDF) [file pgen.1004513.s006.pdf]

Table S4. The PI3K pathway and PLC genes are not involved in embryonic PCD.

A. Mutants defective in the PI3K pathway have normal numbers of cell corpses.

	Number of cell corpses[a]				
Genotype	Comma	1.5-fold	2-fold	3-fold	4-fold
Wild type (at 20℃)	10.0 ± 1.6	11.4 ± 1.5	10.0 ± 1.9	2.1 ± 1.7	0.5 ± 0.7
Wild type (at 25℃)	10.1 ± 2.5	11.1 ± 2.1	10.0 ± 1.9	2.2 ± 1.9	0.5 ± 0.7
aap-1(ok282)	10.3 ± 1.4	11.8 ± 2.0	10.6 ± 1.6	2.3 ± 1.2	0.6 ± 0.7
aap-1(m889)	10.3 ± 1.8	11.4 ± 1.9	10.5 ± 1.9	2.5 ± 1.3	0.7 ± 0.6
age-2(yw23); age-1(hx546) (at 25℃)[b]	10.4 ± 1.9	11.2 ± 1.0	10.5 ± 1.6	2.9 ± 1.1	0.5 ± 0.6
daf-16(m26); age-1(m333)	10.1 ± 2.2	11.1 ± 1.6	10.0 ± 1.7	2.1 ± 1.0	0.5 ± 0.6

B. Mutants defective in PLC genes have normal numbers of cell corpses.

	Number of cell corpses[a]				
Genotype	Comma	1.5-fold	2-fold	3-fold	4-fold
plc-1(rx1)[c]	10.5 ± 1.3	11.0 ± 2.3	10.5 ± 2.3	2.5 ± 2.0	0.7 ± 0.8
plc-2(ok1761)	10.0 ± 2.4	10.8 ± 1.9	10.5 ± 2.8	2.0 ± 1.1	0.6 ± 0.7
plc-3(tm1340)[d]	10.4 ± 2.0	11.7 ± 1.9	10.9 ± 1.9	2.7 ± 1.6	0.5 ± 0.6
plc-4(ok1215)	10.6 ± 1.8	11.9 ± 2.6	10.5 ± 1.6	2.3 ± 1.6	0.7 ± 0.7

[a]Cell corpses scored at the indicated developmental stages are presented as the mean ± SD (n≥30).

[b]The *age-2(yw23); age-1(hx546)* strain is temperature-sensitive and its cell corpse profile was scored at 25℃.

[c]GFP-negative progeny of *plc-1(rx1); kfEx2[plc-1(+); sur-5::gfp]* adult worms.

[d]GFP-negative progeny of *plc-3(tm1340)/mIn1[mIs14 dpy-10(e128)]* adult worms.
