# Supplementary material for: LIN-3/EGF Promotes the Programmed Cell Death of Specific Cells in Caenorhabditis elegans by Transcriptional Activation of the Pro-apoptotic Gene egl-1
Source: PLoS Genet. 2014 Aug 21;10(8):e1004513. doi: 10.1371/journal.pgen.1004513 (PMC4140636; doi:10.1371/journal.pgen.1004513)
Supplement: Table S5 — The lin-1 mutants, but not lin-31 mutants, have reduced numbers of cell corpses. (PDF) [file pgen.1004513.s007.pdf]

Table S5. *lin-1* mutants, but not *lin-31* mutants, have reduced numbers of cell corpses.

	Number of cell corpses[a]				
Genotype	Comma	1.5-fold	2-fold	3-fold	4-fold
Wild-type	10.0 ± 1.6	11.4 ± 1.5	10.0 ± 1.9	2.1 ± 1.7	0.5 ± 0.7
lin-3(e1417)	6.8 ± 2.0**	8.8 ± 2.3**	6.3 ± 2.4**	1.5 ± 1.8	0.2 ± 0.6
lin-1(sy254)	6.7 ± 2.6**	8.9 ± 2.0**	6.3 ± 2.3**	1.5 ± 1.6	0.3 ± 0.6
lin-1(n1047)	6.7 ± 1.7**	9.0 ± 3.0**	6.7 ± 2.3**	1.5 ± 1.3	0.5 ± 0.6
lin-1(n303)	7.7 ± 1.8**	8.5 ± 2.2**	7.0 ± 2.5**	1.8 ± 1.7	0.4 ± 0.7
lin-1(n1761)	8.0 ± 2.4**	8.4 ± 2.5**	5.8 ± 2.8**	1.6 ± 2.0	0.3 ± 0.5
lin-31(n301)	9.6 ± 2.4	10.8 ± 3.3	10.2 ± 2.7	2.3 ± 1.9	0.5 ± 0.7
lin-31(n1053)	10.4 ± 1.6	11.4 ± 2.3	10.7 ± 2.1	2.3 ± 1.3	0.7 ± 0.7
lin-31(gk569)	10.1 ± 1.9	11.5 ± 2.4	10.6 ± 2.4	2.0 ± 1.8	0.7 ± 0.8
lin-1(sy254) lin-3(e1417)	7.2 ± 1.5**	9.3 ± 1.4**	7.1 ± 1.3**	2.3 ± 1.8	0.4 ± 0.6

[a]Cell corpses scored at the indicated developmental stages are presented as the mean ± SD. (n≥30). Mutant embryos were compared to wild-type (**P*<0.05 and ***P*<0.001, two-tailed *t* test). The cell corpse numbers of *lin-1(sy254) lin-3(e1417)* double mutants showed no significant difference when compared to the *lin-1(sy254)* or *lin-3(e1417)* single mutant (*P*>0.05, two-tailed *t* test).
